# The use of placebo in randomised surgical clinical trials

**DOI:** 10.1186/1745-6215-14-S1-P38

**Published:** 2013-11-29

**Authors:** Karolina Wartolowska, Andrew Judge, Benjamin Dean, Ines Rombach, Julian Savulescu, David Beard, Andrew Carr

**Affiliations:** 1 Nuffield Department Nuffield Department of Orthopaedics, Rheumatology and Musculoskeletal Sciences, Oxford, UK; 2 MRC Lifecourse Epidemiology Unit, Southampton, UK; 3 Oxford Uehiro Centre for Practical Ethics, Oxford, UK

## Background

Placebo has an established role in pharmacological trials but is rarely used to examine the efficacy of surgical interventions. The aim of this study was to analyse the efficacy of the crucial surgical element in comparison to the placebo arm in randomised trials investigating surgical procedures.

## Methods

We searched MEDLINE, EMBASE, and the Cochrane Controlled Trials Register, from their beginning to September 2012, and screened the references of the already included studies. We reviewed randomised clinical trials comparing surgery with a placebo intervention. Surgery was defined as any procedure that changes the anatomy and requires a skin incision or the use of endoscopic techniques; dental studies were excluded. We did not limit the search to any particular type of intervention or condition.

## Results

We identified 96 relevant reports corresponding to 74 unique studies. Fifty-two trials were included into the meta-analysis. In three quarters of the studies (38/52) an improvement was reported in both arms and only in half of the trials (25/52) the active treatment was more effective than the placebo. The effect was generally in favour of the active treatment but in many trials the difference was statistically significant only for some outcomes.

## Conclusions

Placebo-controlled randomised trials have a significant value in assessing the absolute efficacy of surgery, especially when the outcomes are subjective and the likelihood of a placebo effect is high. Interventions that are truly effective and superior to placebo should be encouraged and the procedures that are not more effective than placebo should not be performed.

